# Genomic and transcriptomic analysis of yield enhancement mechanism of lipopeptide iturin W by tryptone supplementation

**DOI:** 10.1128/spectrum.03247-25

**Published:** 2025-11-24

**Authors:** Jiao Chen, Yingyu Ji, Chaomin Sun, Shimei Wu

**Affiliations:** 1 College of Life Sciences, Qingdao University 12593, Qingdao, China; 2 CAS Key Laboratory of Experimental Marine Biology, Institute of Oceanology, Chinese Academy of Sciences 53014, Qingdao, China; 3 Laboratory for Marine Biology and Biotechnology, Qingdao National Laboratory for Marine Science and Technology 474988 , Qingdao, China; 4 Center of Ocean Mega-Science, Chinese Academy of Sciences, Qingdao, China; University of Minnesota Twin Cities, St. Paul, Minnesota, USA

**Keywords:** genomic, transcriptomic, biosynthesis, *Bacillus*, lipopeptide

## Abstract

In our previous studies, a novel lipopeptide, iturin W, exhibiting high antifungal activity, was discovered from *Bacillus* sp. wsm-1. To enhance its production, the effects of different carbon and nitrogen sources were detected, and only supplementation of tryptone significantly increased the yield of iturin W. In this study, the biosynthetic gene cluster of iturin W was identified through antiSMASH analysis of the *Bacillus* sp. wsm-1 genome, followed by gene knockout validation. The cluster comprises four open reading frames: *ituW-D*, *ituW-A*, *ituW-B*, and *ituW-C*. To elucidate the enhancement mechanism of iturin W production by tryptone supplementation, transcriptomic analysis was conducted. The results revealed that tryptone supplementation significantly upregulated the gene cluster responsible for iturin W biosynthesis, along with genes associated with the TCA cycle, ribosome function, pyruvate metabolism, fatty acid metabolism, and amino acid metabolism. Additionally, the quorum sensing system Rap-Phr and several key genes were closely linked to iturin W biosynthesis. Notably, the overexpression of BslA and cytochrome P450 gene together resulted in a more than threefold increase in iturin W production. Overall, this study not only provides insights into the yield enhancement mechanism of iturin W in response to tryptone supplementation but also identifies key enzymes and potential regulators involved in its biosynthesis, providing the foundation for the yield improvement of lipopeptide in the future.

**IMPORTANCE:**

Lipopeptides exhibit a variety of biological activities and hold promising potential for various applications, but their practical use is largely limited by low yields. Medium optimization is a fundamental approach to enhance the yield of lipopeptide, and numerous studies have demonstrated that optimizing the medium can increase lipopeptide production to varying degrees. However, the underlying mechanisms driving these improvements have seldom been explored. In this study, the yield enhancement mechanism of iturin W by supplementation of tryptone has been investigated by transcriptomic analysis. The result not only elucidated the detailed pathway underlying iturin W production improvement but also discovered key enzymes and potential regulators involved in its biosynthesis. Overexpression of these genes significantly increased iturin W production. Collectively, our findings provide a valuable foundation for improving lipopeptide yields in the future.

## INTRODUCTION

Lipopeptides, secondary metabolites of microorganisms, consist of hydrophobic long alkyl chains (13–19 carbon atoms) and hydrophilic polypeptides composed of 7–10 amino acids. Based on their peptide chain and fatty acid structures, lipopeptides produced by *Bacillus* species are primarily classified into three categories: iturins, fengycins, and surfactins (1). Due to their amphipathic characteristics, lipopeptides are capable of forming pores and ion channels in cell membranes, which enable them to exhibit a broad range of biological activities, including antifungal, antibacterial, antiviral, and anticancer properties. As a result, they hold significant potential for application in agriculture, pharmaceuticals, and the food industry (2). However, the low yield of lipopeptides remains a major challenge, significantly limiting their practical applications.

Lipopeptides are biosynthesized by multi-enzyme complexes known as nonribosomal peptide synthetases (NRPSs) or hybrid polyketide synthases and nonribosomal peptide synthetases (PKSs/NRPSs) (3). The biosynthesis of lipopeptide in *Bacillus* species is tightly regulated by quorum sensing system, such as the ComQXPA system and the Rap-Phr system (4, 5), as well as by other regulatory factors, including DegU, DegQ, SigA, AbrB, and CodY (6, 7). To enhance lipopeptide production, various genetic engineering methods targeting the biosynthetic pathway have been explored (8, 9). For example, overexpressing *comA* and *sigA* has been shown to significantly increase iturin A production, while knocking out *rapC* can boost the yield of the lipopeptide surfactin (10, 11). Additionally, optimizing culture conditions is another effective method to improve lipopeptide production (12). Nitrogen sources, a fundamental energy source for biological growth, are also essential for lipopeptide biosynthesis. Previous studies have demonstrated that supplementing various nitrogen sources can enhance lipopeptide production to varying degrees. However, the underlying mechanisms remain poorly understood (13–15). Investigating these mechanisms could contribute to identify novel key enzymes and regulatory factors, providing a scientific foundation for improving lipopeptide yields.

In our previous study, a novel lipopeptide, iturin W, was isolated from *Bacillus* sp. wsm-1, a marine strain derived from deep-sea cold seep sediments, and exhibited strong antifungal activity against a range of plant pathogens (16). We also investigated the effects of different carbon and nitrogen sources on iturin W production, finding that only tryptone supplementation significantly increased its yield. To uncover the underlying mechanisms, genomic and transcriptomic analyses were conducted in this study, and genes involved in production of iturin W were explored through overexpression, hoping to provide a theoretical foundation for further improving lipopeptide production in the future.

## RESULTS

### Genomic analysis and identification of iturin W biosynthetic gene cluster

To determine the biosynthetic gene cluster of lipopeptide iturin W, the whole genome of *Bacillus* sp. wsm-1 was fully sequenced. As shown in Table 1, the genome assembly resulted in a complete circular chromosome of 3,929,584 bp, with a GC content of 46.5%. No plasmids were detected. The genome contains 86 amino acid acyl-tRNA synthetase genes corresponding to all 20 amino acids and 3,784 coding sequences (CDSs), with a total length of genes of 3,526,305 bp, which constitutes 89.74% of the whole chromosome. In addition, 142 tandem repeats (TR), 120 minisatellite DNA, and 1 microsatellite DNA were predicted. The genome also contains 15 prophages.

**TABLE 1:** Assembly statistics of the genome of *Bacillus* sp. wsm-1

Category	Parameters
Genome size (bp)	3,929,584
GC content (%)	46.5
Gene number	3,866
CDSs	3,748
Chromosome	1
Gene total length (bp)	3,526,305
Gene average length (bp)	912
Gene length/Genome (%)	89.74
tRNA gene	86
rRNA gene	27
sRNA	9
Minisatellite DNA	120
Microsatellite DNA	1
Prophage	15
TR	142

**Fig 1:**
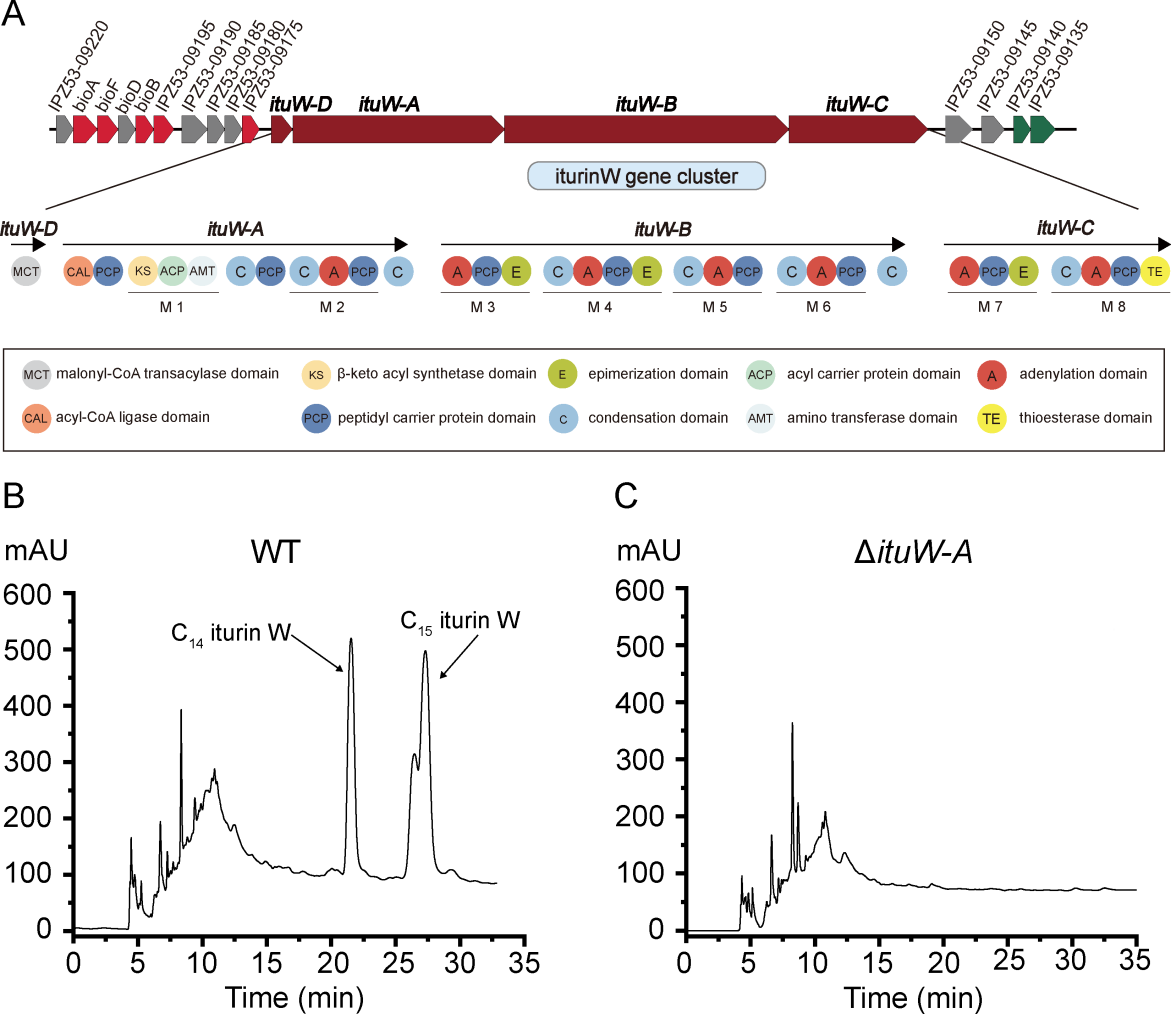
The schematic diagram of iturin W biosynthetic gene cluster (**A**) and HPLC analysis of iturin W production in wild- type *Bacillus* sp. wsm-1 (**B**) and *Bacillus* sp. wsm-1 mutant strain Δ*ituW-A* (**C**).

Based on antiSMASH analysis of the genomic sequence of *Bacillus* sp. wsm-1, a large gene cluster associated with iturin W biosynthesis was obtained (Fig. 1A), consisting of four open reading frames: *ituW-D* (*IPZ53_RS09170*), *ituW-A* (*IPZ53_RS09165*), *ituW-B* (*IPZ53_RS09160*), and *ituW-C* (*IPZ53_RS09155*). BLAST search against the NCBI non-redundant protein database showed that the proteins encoded by *ituW-D* and *ituW-A* shared 98.96% and 98.87% homology, respectively, to the non-ribosomal peptide synthetases BamD and BamA of bacillomycin, while the proteins encoded by *ituW-B* and *ituW-C* exhibited 97.82% and 97.82% homology to non-ribosomal peptide synthetases A and B of iturin A. Further analysis revealed that *ituW-A* contains two modules: module 1, which contains β-ketoacyl synthase domain, acyl carrier protein domain and amino transferase domain, is involved in the formation of β-amino fatty acids chain and the assembly of the amino acid molecule in the peptide segment. Module 2, which contains a typical C domain, A domain, and PCP domain, is responsible for the biosynthesis of the first amino acid. *ItuW-B* contains four modules (M3-M6), responsible for synthesizing four specific amino acids. *ItuW-C* contains two modules (M7, M8), responsible for the biosynthesis of the last two amino acids. The thioesterase (TE) domain in the M8 module contributes to peptide cyclization. Based on the above analysis, we hypothesize that this non-ribosomal biosynthetic gene cluster is responsible for biosynthesis of lipopeptide iturin W.

To confirm the involvement of the predicted gene cluster in the biosynthesis of iturin W, gene *ituW-A* was knocked out in *Bacillus* sp. wsm-1, and HPLC analysis was performed to compare the production of iturin W between the wild-type strain and the *ituW-A* knockout strain. As shown in Fig. 1B, C_14_ and C_15_ iturin W peaks were detected at 20.419 and 25.726 min in the wild-type strain, consistent with our previous report (16). However, no corresponding peaks were observed in the *ituW-A* knockout strain (Fig. 1C), indicating that the *ituW-A* gene is essential for the production of iturin W. These results confirm that the gene cluster composed of *ituW-D*, *ituW-A*, *ituW-B*, and *ituW-C* is closely related to the biosynthesis of iturin W.

### Overview of differentially expressed genes in response to tryptone supplementation

**Fig 2:**
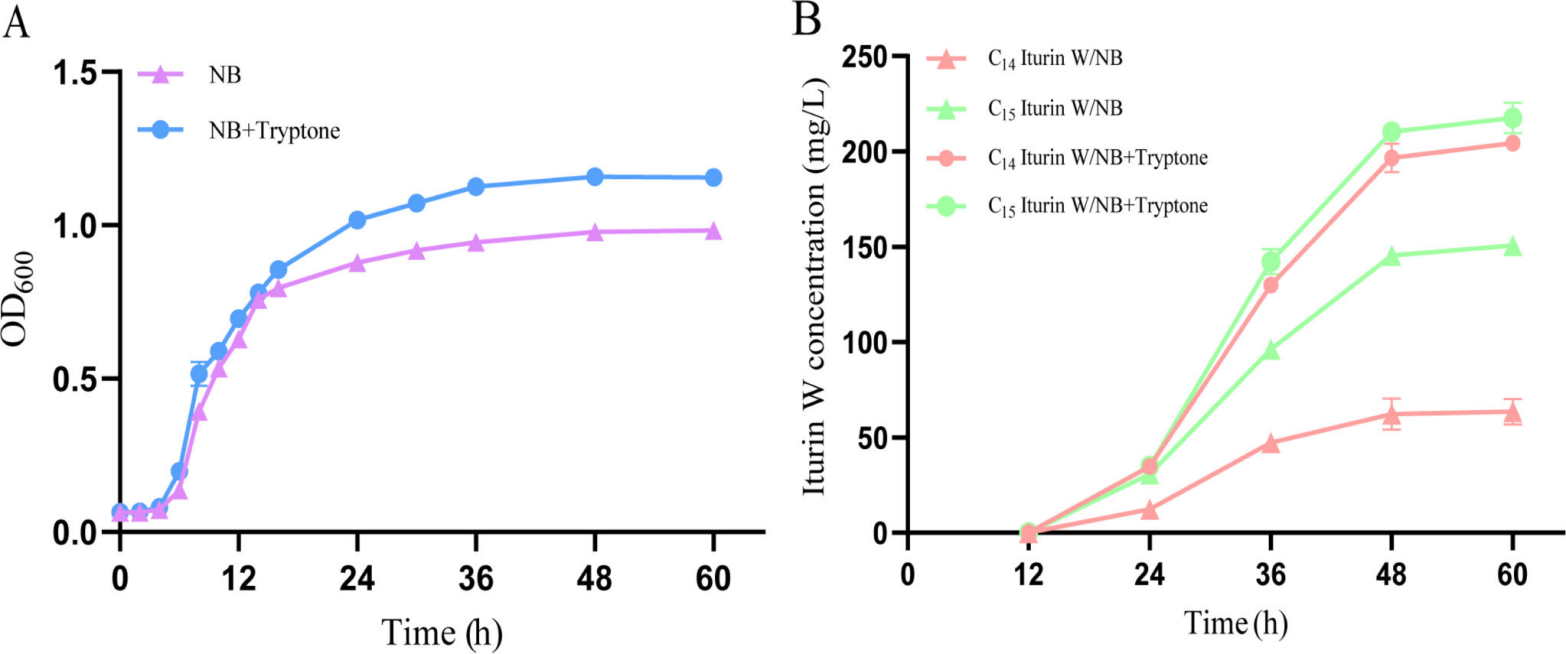
The growth curve (**A**) and iturin W production (**B**) of *Bacillus* sp. wsm-1 with and without tryptone supplementation.

In our previous study, tryptone supplementation was found to significantly enhance the yield of iturin W in *Bacillus* sp. wsm-1 (16). To investigate the underlying mechanism, we analyzed the growth curve of *Bacillus* sp. wsm-1 and the production of iturin W, with and without tryptone supplementation. The results indicated similar growth patterns, with only a slight increase in cell density observed under tryptone supplementation (Fig. 2A). For iturin W production, small amounts were detected at 24 h, with production reaching its peak at 48 h. Notably, tryptone supplementation significantly enhanced iturin W production, particularly C_14_ iturin W (Fig. 2B).

**Fig 3:**
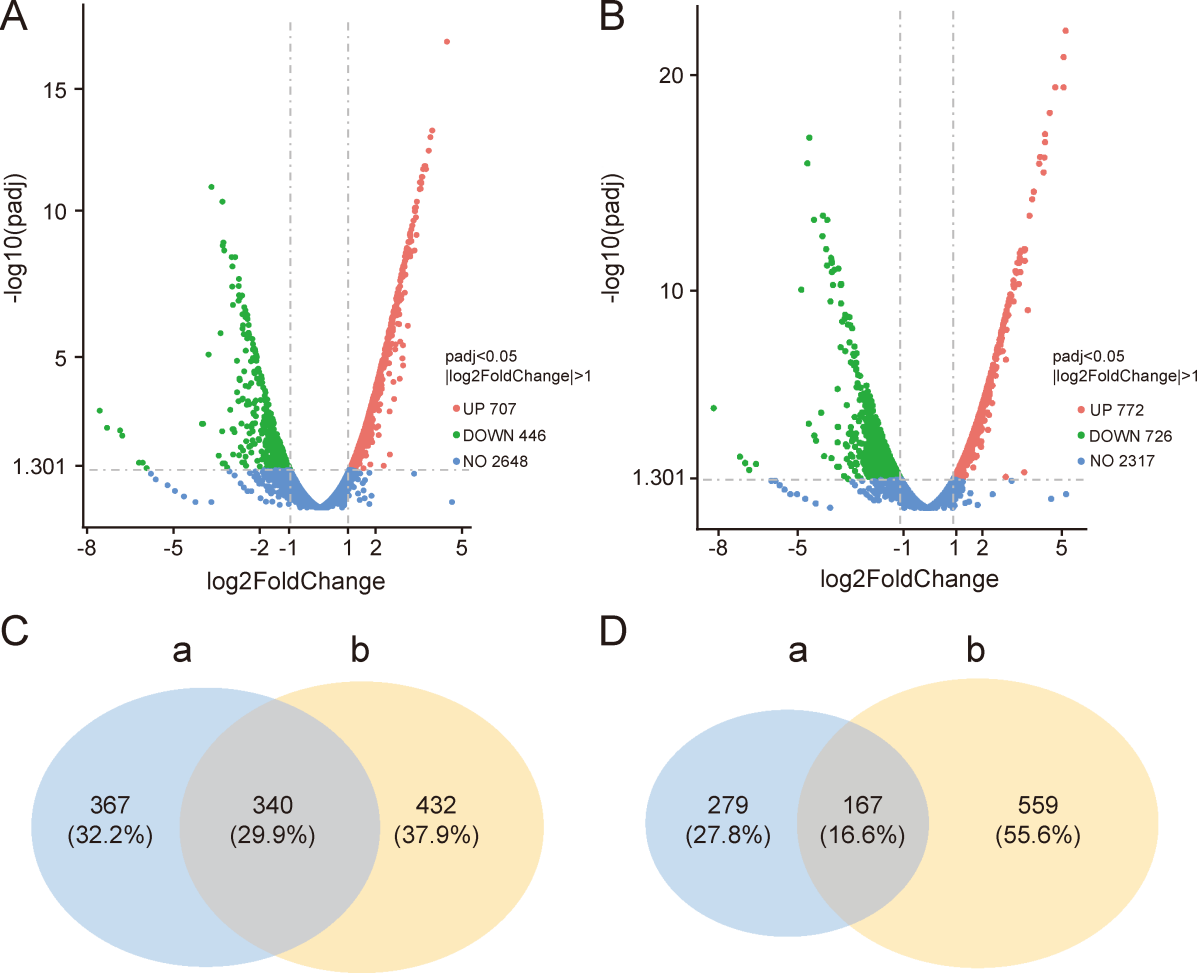
The volcano plots of the DEGs in *Bacillus* sp. wsm-1 in response to tryptone supplementation after 24 h (**A**) and 48 h (**B**) of incubation, and the Venn diagrams of up-regulated DEGs (**C**) and down-regulated DEGs (**D**) between groups incubated for 24 h and 48 h, respectively. “a” indicates DEGs in groups with or without tryptone supplementation after incubation for 24 h. “b” indicates DEGs in groups with or without tryptone supplementation after incubation for 48 h.

Based on the above results, a comparative transcriptomic analysis was conducted on *Bacillus* sp. wsm-1 cultures, supplemented with or without tryptone, after 24 and 48 h of incubation. After 24 h of incubation, a total of 1,153 differentially expressed genes (DEGs) were identified in the tryptone-supplemented group compared to the control group without tryptone supplementation. Among these, 707 DEGs were upregulated, and 446 DEGs were downregulated (Fig. 3A). A greater number of DEGs were observed after 48 h of incubation (Fig. 3B). As shown in the venn diagram, 340 genes were shared among the upregulated DEGs after 24 h and 48 h of incubation (Fig. 3C), while 167 genes were shared among the downregulated DEGs (Fig. 3D).

**Fig 4:**
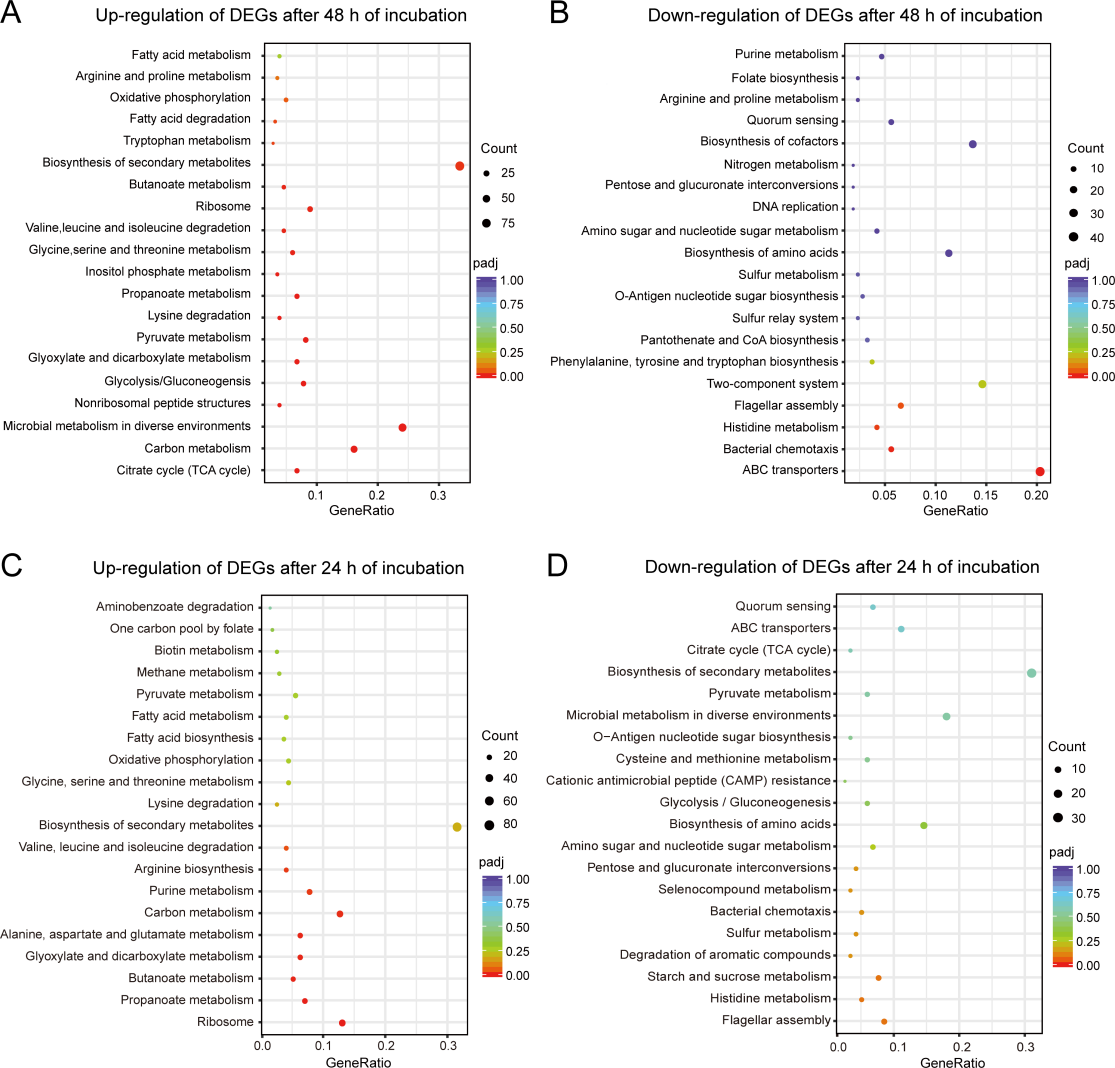
Scatter plot of enriched KEGG pathways statistics in response to tryptone supplementation for upregulated DEGs (**A**) and downregulated DEGs (**B**) after incubation for 48 h, and upregulated DEGs (**C**) and downregulated DEGs (**D**) after incubation for 24 h, respectively. Gene ratio: the ratio of the number of differential genes to the total number of differential genes noted on the KEGG pathway number. Count is the number of differential genes annotated on KEGG pathway, and padj is the *P* value after multiple hypothesis test. The size of the dots indicates the number of expressed genes in the pathways, and the color of the dots represents the *P* value of the pathway.

Furthermore, KEGG pathway enrichment was also analyzed for the DEGs after 24 and 48 h of incubation, respectively. For the 48 h incubation group, the upregulated genes were primarily associated with citrate cycle (TCA cycle), ribosome, pyruvate metabolism, fatty acid metabolism, and amino acid metabolism (Fig. 4A). The downregulated genes were mainly involved in flagellar assembly, histidine metabolism, bacterial chemotaxis, and ABC transporters (Fig. 4B). For the 24 h incubation group, the KEGG pathway analysis showed similar results to the 48 h group (Fig. 4C and D).

### DEGs involved in fatty acid biosynthesis in response to tryptone supplementation

**Fig 5:**
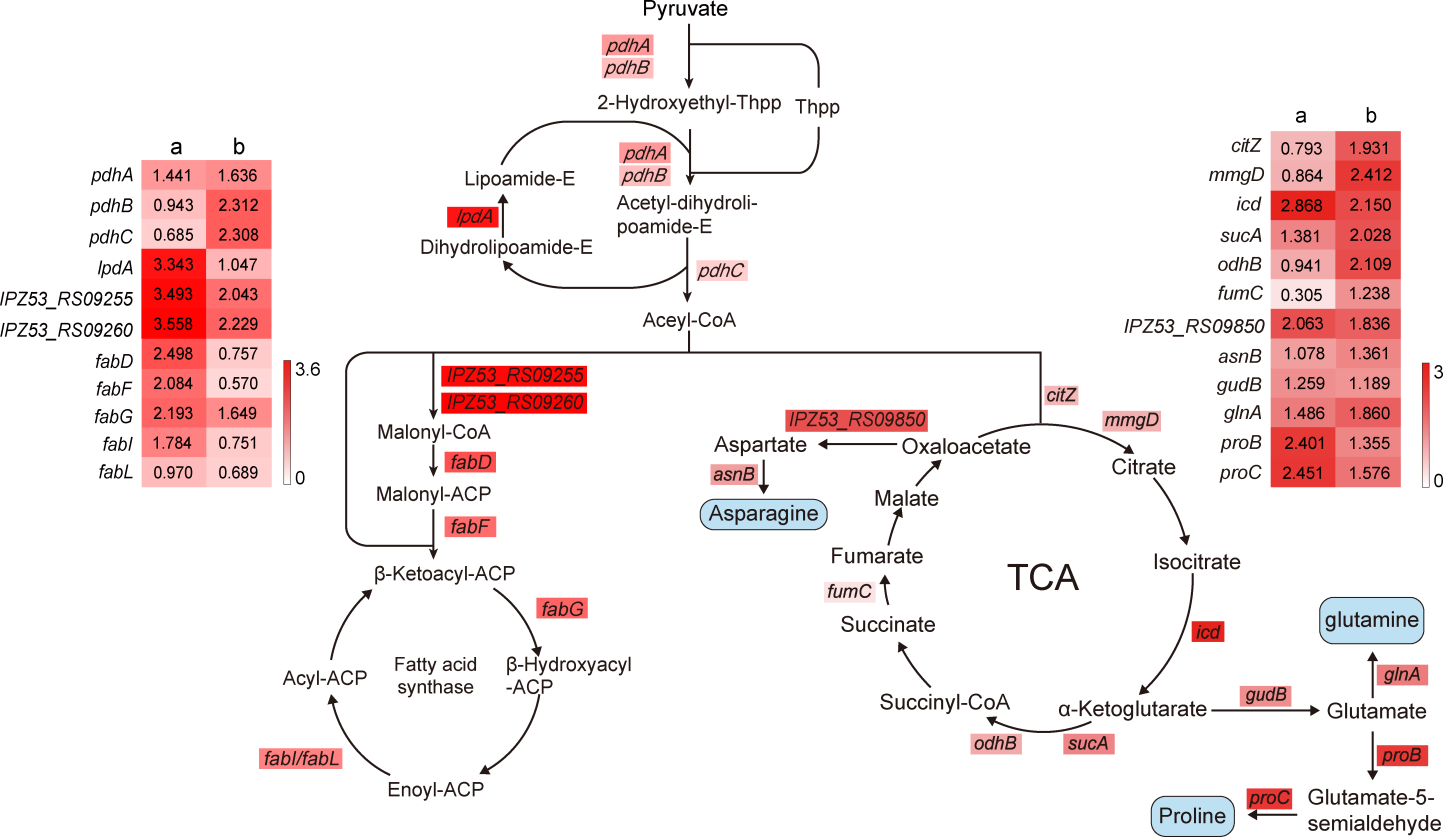
The pathway diagram of fatty acid, amino acids biosynthesis, and TCA cycle with heat map of corresponding DEGs. The a and b indicate the relative expression of DEGs in groups with tryptone supplementation vs that in groups without tryptone supplementation after incubation for 24 h and 48 h, respectively. The log_2_fold change was colored and represents the ratio of gene expression level between the groups with and without tryptone supplementation.

For the fatty acid chain is an important part of lipopeptide iturin W, the expression levels of genes related to the fatty acid biosynthesis pathway were analyzed in details in our study. As shown in Fig. 5, the genes *pdhA, pdhB,* and *pdhC,* which are involved in biosynthesis of acetyl-CoA, were all upregulated. Additionally, the expression of the gene *IPZ53_RS09255*, which encodes acetyl-CoA carboxylase biotin carboxyl carrier protein subunit, and the gene *IPZ53_RS09260*, encoding acetyl-CoA carboxylase biotin carboxylase subunit, were also upregulated. These genes play crucial roles in converting acetyl-CoA to malonyl-CoA (17, 18). Moreover, the expression of the gene *fabD*, *fabF*, *fabG,* and *FabI/ Fab* was also increased, which are essential enzymes in the fatty acid biosynthesis pathway (19–21). Thus, following tryptone supplementation, the upregulation of most genes involved in fatty acid biosynthesis facilitated the formation of the fatty acid chain in lipopeptide iturin W.

### DEGs involved in TCA cycle and amino acid biosynthesis in response to tryptone supplementation

Since iturin W contains five amino acids: Asn, Gln, Ser, Pro, and Tyr, the expression levels of genes involved in biosynthesis of these amino acids were further analyzed. As shown in Fig. 5, many genes related to the TCA cycle, such as gene *mmgD, icd*, *sucA, odhB,* and *fumC*, were upregulated after tryptone supplementation. The biosynthesis of Asn and Gln, derived from oxaloacetate and α-ketoglutarate in the TCA cycle, was also upregulated. For Asn biosynthesis, oxaloacetate was first catalyzed to Asp by aspartate aminotransferase, then further catalyzed to Asn by asparagine synthase. The corresponding genes *IPZ53_RS09850* and *asnB* were upregulated after tryptone supplementation. For Gln biosynthesis, α-ketoglutarate was first catalyzed by glutamate dehydrogenase (encoded by *gudB*) and then converted to Gln by glutamine synthetase (encoded by *glnA*). These genes were also upregulated. For Pro biosynthesis, the genes *proB,* encoding glutamate-5-kinase, and *proC,* encoding pyrroline-5-carboxylate reductase, were also upregulated after tryptone supplementation.

### Analysis of the significantly changed DEGs in response to tryptone supplementation

To investigate why tryptone supplementation increased the production of iturin W, dramatically changed DEGs, potentially related to nitrogen utilization and production of lipopeptide based on previous reports, were analyzed in details. As shown in Table 2, the expression of the gene *IPZ53_RS04215*, encoding a kind of serine protease trypsin, was upregulated after 24 h and 48 h of incubation, which may contribute to nitrogen degradation. Additionally, the genes *glnA* encoding glutamine synthase and *glsA* encoding glutaminase A, both involved in nitrogen assimilation, were also notably upregulated. Therefore, after tryptone supplementation, a range of genes associated with nitrogen degradation and assimilation were upregulated.

**TABLE 2:** Significant changed DEGs based on transcriptomic analysis

Gene name	24 h log_2_foldchange	48 h log_2_foldchange	Gene description
*ituW-A*	1.198	2.498	Non-ribosomal peptide synthetase
*ituW-B*	1.169	2.866	Non-ribosomal peptide synthetase
*ituW-C*	0.935	2.405	Non-ribosomal peptide synthetase
*ituW-D*	0.646	2.014	Bacillomycin D biosynthesis malonyl-CoA transacylase BamD
*IPZ53_RS04215*	2.618	4.639	Serine protease && PF00089: Trypsin
*glnA*	1.486	1.860	Glutamine synthase
*glsA*	2.631	1.286	Glutaminase A
*IPZ53_RS17575*	3.331	1.885	PhrC/PhrF family phosphatase-inhibitory pheromone
*IPZ53_RS09195*	2.683	1.780	Cytochrome P450
*IPZ53_RS11070*	1.281	3.681	Cytochrome P450
*IPZ53_RS14295*	2.107	4.271	Biofilm surface layer hydrophobin BslA
*IPZ53_RS04210*	2.641	4.846	DUF2606 family protein with unknown function
*IPZ53_RS09530*	3.656	2.171	LysM peptidoglycan-binding domain containing protein with unknown function
*IPZ53_RS02295*	1.045	5.242	Hypothetical protein
*spoIIIAA*	3.086	1.920	Stage III sporulation protein AA
*spoIID*	2.233	2.084	Stage II sporulation protein D
*spoIIM*	1.967	1.164	Stage II sporulation protein M
*cotJC*	3.279	1.940	Spore coat protein CotJC
*sigK*	2.037	2.126	RNA polymerase sporulation sigma factor SigK

The biosynthesis gene cluster of iturin W contains four large open reading frames: *ituW-A*, *ituW-B*, *ituW-C*, and *ituW-D*. Compared to the control group without tryptone supplementation, the expression levels of these genes were significantly upregulated in the tryptone-supplemented groups. Furthermore, gene involved in quorum sensing system, such as gene *IPZ53_RS17575* encoding PhrC/PhrF family pheromone, was also upregulated. The expression levels of genes *IPZ53_RS09195* and *IPZ53_RS11070*, encoding cytochrome P450, and *IPZ53_RS14295*, encoding biofilm surface layer hydrophobin BslA, showed varying degrees of up-regulation. In addition, genes with unknown function, such as *IPZ53_RS04210*, *IPZ53_RS09530*, and *IPZ53_RS02295*, also exhibited significant increases in expression.

Remarkably, tryptone supplementation also upregulated the expression of genes associated with spore formation, such as genes *spoIIIAA*, *spoIID,* and *spoIIM*, which are involved in maintaining the integrity and location of prespores (22, 23), as well as *cotJC,* which is related to coat formation and assembly (24). Additionally, the regulatory factor *sigK* was dramatically upregulated. Thus, tryptone supplementation not only enhanced the expression of genes involved in the biosynthesis of iturin W but also altered the expression of genes linked to spore formation. In line with previous reports showing that the sporulation regulator Spo0A positively regulates the biosynthesis of lipopeptides like lipopeptide bacillomycin D and polymyxin (25, 26), we speculated that lipopeptide production may be closely related to spore formation.

### qRT-PCR verification of the transcriptomic analysis

**Fig 6:**
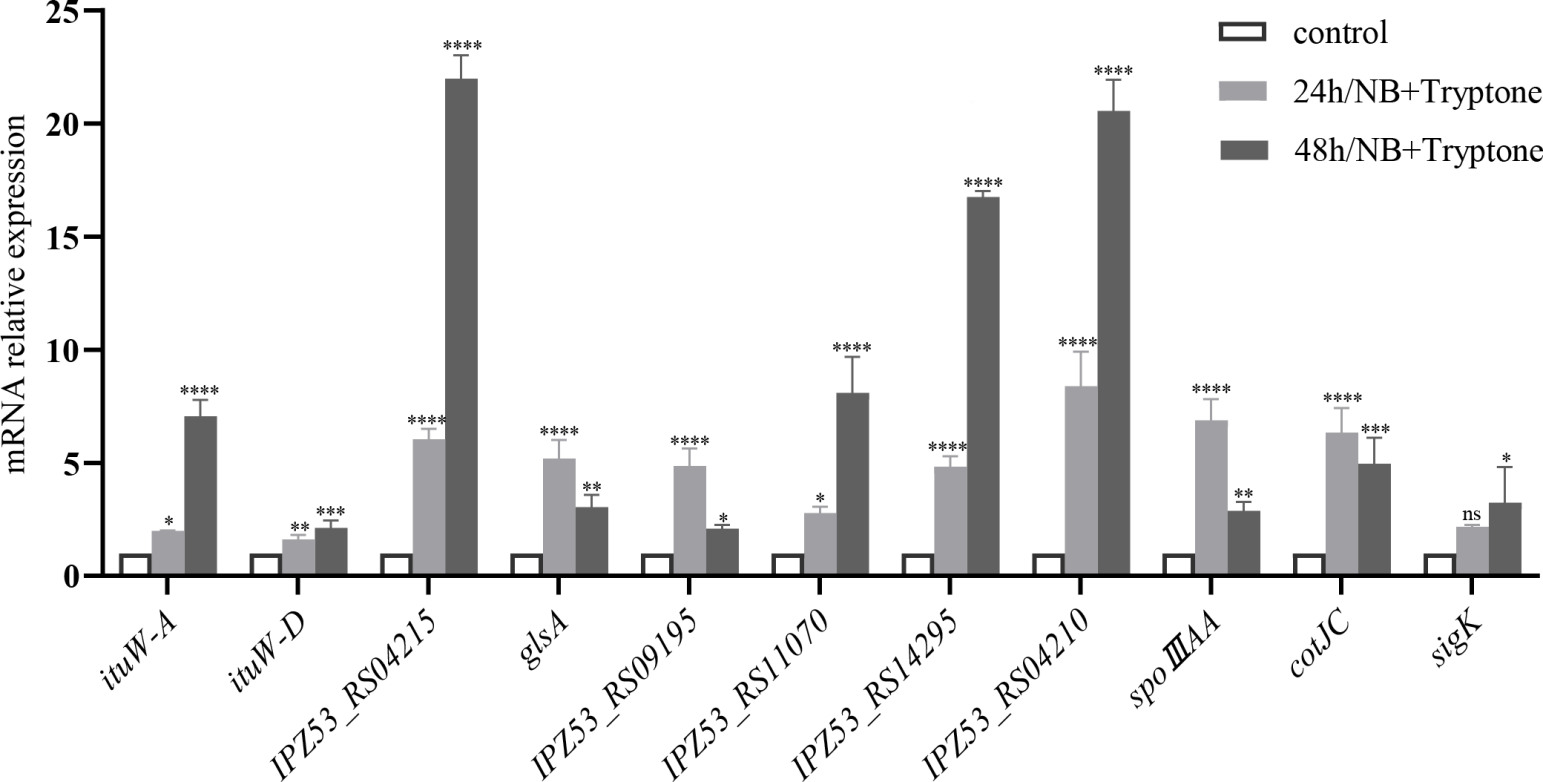
Relative expression of *ituW-A, ituW-D, IPZ53_RS04215, glsA, IPZ53_RS09195, IPZ53_RS11070, IPZ53_RS14295, IPZ53_RS04210, spoIIIAA, cotJC,* and *sigK* in *Bacillus* sp. wsm-1 cultured in NB medium with/without tryptone supplementation at 24 h and 48 h.*P < 0.05, **P < 0.01, ****P < 0.001.

To provide experimental validation of the RNA-seq results, qRT-PCR was performed to measure the expression of a subset of DEGs in response to tryptone supplementation at 24 and 48 h. As shown in Fig. 6, the genes related to biofilm surface layer hydrophobin BslA (*IPZ53_RS14295*), Cytochrome P450 (*IPZ53_RS09195* and *IPZ53_RS11070*), glutaminase A (*glsA*), and a LysM peptidoglycan-binding domain-containing protein of unknown function (*IPZ53_RS09530*) were significantly upregulated at both 24 and 48 h after tryptone supplementation. The high concordance between the qRT-PCR and RNA-seq results demonstrates the reliability of our transcriptome data set.

### Verification of the relationship between significantly changed DEGs and production of iturin W

**Fig 7:**
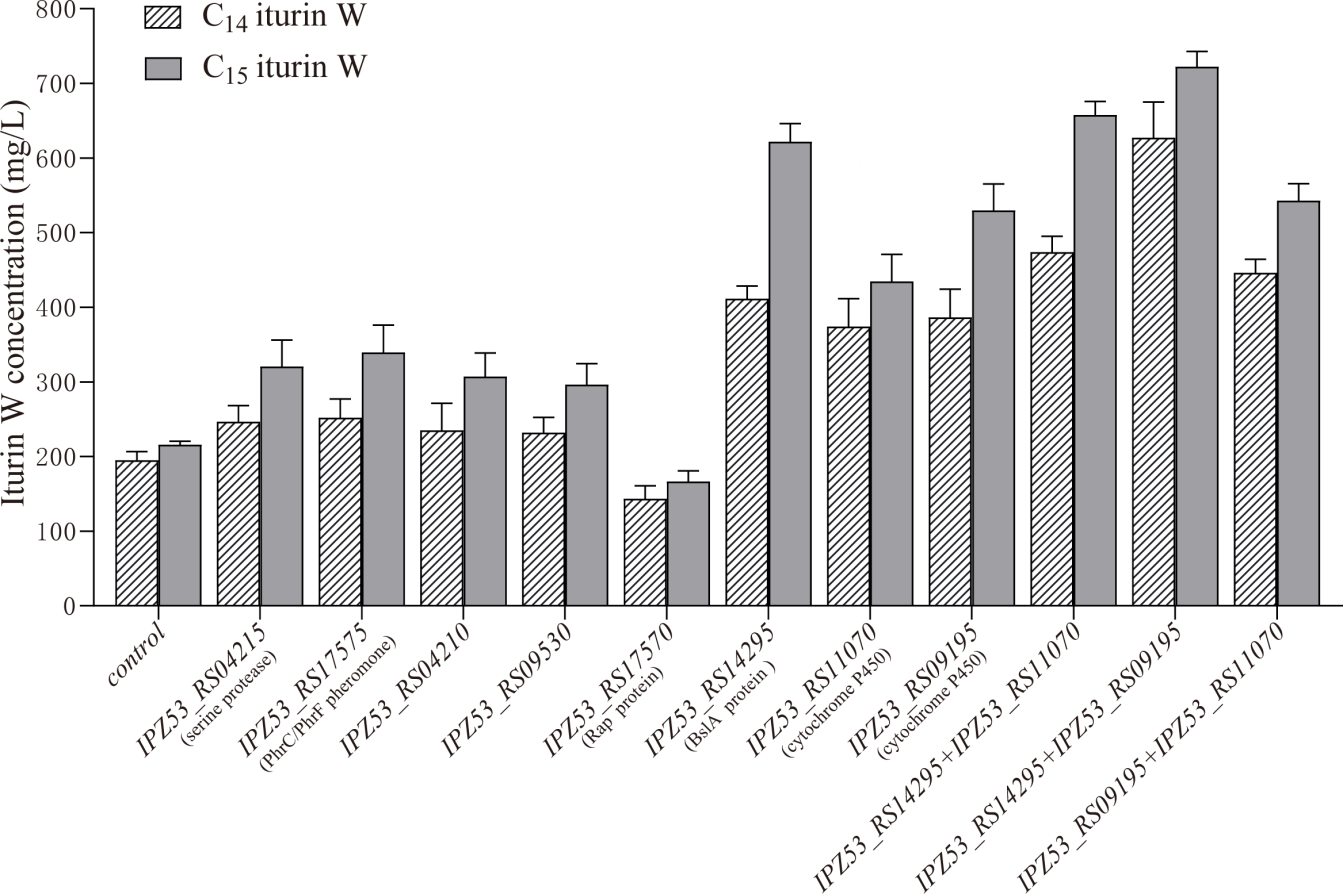
Production of C_14_ iturin W and C_15_ iturin W in wild type strain *Bacillus* sp. wsm-1 and corresponding DEGs overexpressed strains. “control” indicates the wild type strain *Bacillus* sp. wsm-1 containing the empty vector pMarAΔ*Himar1*, and the “gene name” indicates corresponding genes overexpressed by plasmid pMarAΔ*Himar1* in *Bacillus* sp. wsm-1.

To verify the relationship between significantly changed DEGs and the production of iturin W, some key DEGs in Table 2, potentially linked to nitrogen utilization and production of lipopeptide based on previous reports, were overexpressed in *Bacillus* sp. wsm-1. The production of iturin W was then assessed under tryptone supplementation. As shown in Fig. 7, compared to the control group of *Bacillus* sp. wsm-1, the overexpression of gene *IPZ53_RS14295,* which encodes biofilm surface layer hydrophobin BslA, resulted in the production of iturin W increased about 2.11 times for C_14_ iturin W and 2.88 times for C_15_ iturin W. Furthermore, the overexpression of gene *IPZ53_RS11070* and *IPZ53_RS09195,* which encode cytochrome P450, also resulted in a more than twofold increase in iturin W production. In addition, the overexpression of gene *IPZ53_S04215* encoding a kind of serine protease trypsin; *IPZ53_RS17575* encoding the PhrC/PhrF family pheromone; *IPZ53_RS04210* encoding a DUF2606 family protein with unknown function; and *IPZ53_RS09530* encoding a LysM peptidoglycan-binding domain-containing protein, all contributed to varying degrees of increased production of C14 and C15 iturin W. However, the overexpression of *IPZ53_RS17570*, which encodes Rap family tetratricopeptide repeat protein, resulted in a decrease in iturin W production. Therefore, these genes appear to be closely associated with the enhanced production of iturin W in response to tryptone supplementation, highlighting their potential roles in nitrogen utilization and lipopeptide synthesis.

To further explore the impact of overexpressing multiple genes, both the BslA encoding gene *IPZ53_RS14295* and the cytochrome P450 encoding genes *IPZ53_RS11070* and *IPZ53_RS09195* were overexpressed together. As shown in Fig. 7, co-overexpression of *IPZ53_RS14295* (BslA) and *IPZ53_RS09195* (cytochrome P450) resulted in a more than three-fold increase in iturin W production. In contrast, co-overexpression of *IPZ53_RS14295* (BslA) and *IPZ53_RS11070* (cytochrome P450) led to only a slight increase in iturin W production compared to the overexpression of *IPZ53_RS14295* alone. Additionally, co-overexpression of the two cytochrome P450 genes, *IPZ53_RS11070* and *IPZ53_RS09195*, did not yield a greater increase in iturin W production compared to the overexpression of them alone. These results suggest that BslA encoding gene *IPZ53_RS14295* and cytochrome P450 encoding gene *IPZ53_RS09195* are more important for iturin W production.

## DISCUSSION

Lipopeptides are a promising alternative to antibiotics, but their low yield has become a major problem hindering their widespread application. Previous studies have demonstrated that supplementing various nitrogen sources can enhance lipopeptide production to varying degrees although the underlying mechanisms still require further investigation (13–15). In this study, we explored the mechanism by which tryptone supplementation enhances the production of the lipopeptide iturin W. Following tryptone supplementation, a series of genes related to nitrogen degradation and assimilation were upregulated, potentially improving nitrogen utilization and promoting cell growth. Additionally, genes involved in fatty acid biosynthesis, the TCA cycle, and amino acid metabolism were obviously upregulated, which may provide more precursors for iturin W production. Notably, the expression levels of NRPSs involved in iturin W biosynthesis were also markedly increased, leading to a substantial boost in iturin W production.

The quorum sensing system Rap-Phr, composed of response regulator aspartate phosphatase (Rap) and its inhibitory PhrC/PhrF family pheromone, plays a crucial role in complex regulatory network of *Bacillus*, influencing processes such as regulating sporulation and biofilm formation (27, 28). Recent studies have shown that the production of the lipopeptide surfactin is regulated by the Rap-Phr system (5), and knockout of *rapC* enhances the production of lipopeptide bacillomycin D (29). In this study, the gene *IPZ53_RS17575,* encoding a PhrC/PhrF family pheromone, was upregulated in response to tryptone supplementation. Overexpression of *IPZ53_RS17575* led to an increase in iturin W production. Conversely, overexpression of *IPZ53_RS17570*, which encodes a Rap family tetratricopeptide repeat protein, resulted in reduced iturin W production. These findings suggest that the production of iturin W is regulated by the Rap-Phr quorum sensing system, consistent with previous reports (30).

Bacteria commonly form biofilms, where they live embedded within a protective matrix. One key component of biofilm formation is the surface layer hydrophobin BslA, which plays a critical role in biofilm development (31). In our study, the expression of *IPZ53_RS14295*, which encodes biofilm surface layer hydrophobin BslA, was significantly upregulated in response to tryptone supplementation. Overexpression of *IPZ53_RS14295* resulted in more than a two-fold increase in iturin W production, suggesting that hydrophobin BslA is closely associated with the enhanced production of iturin W under tryptone supplementation. Notably, no biofilm was detected under the condition of iturin W production, indicating that the effect of *bslA* on iturin W production is independent of its role in biofilm formation. Additionally, it has been it has been reported that the overexpression of the *cypC* gene, which encodes fatty acid beta-hydroxylating cytochrome P450, increased fengycin production by approximately 46.6% in *Bacillus subtilis* 168 (32). In our study, the expression of gene *IPZ53_RS09195* and *IPZ53_RS11070*, which encode cytochrome P450, was also significantly upregulated with tryptone supplementation. The overexpression of these genes led to a more than twofold increase in iturin W yield, suggesting that cytochrome P450 plays a vital role in the enhancement of iturin W production under tryptone supplementation.

In conclusion, through genomic and transcriptomic analysis of *Bacillus* sp. wsm-1, we identified a biosynthetic gene cluster responsible for iturin W production and clarified the mechanism behind the yield enhancement of iturin W in response to tryptone supplementation. Furthermore, we discovered a series of key enzymes and potential regulators involved in iturin W production, providing a solid foundation for future efforts to improve lipopeptide yields.

## MATERIALS AND METHODS

### Bacterial strain and culture condition

The lipopeptide iturin W producing strain *Bacillus* sp. wsm-1 was usually cultured in nutrient broth (NB) medium (peptone at 10 g/L, beef powder at 3 g/L, NaCl at 5 g/L, distilled water at 1,000 mL, pH adjusted to 7.0) and incubated at 28°C under vigorous agitation at a speed of 150 rpm for 24 h and 48 h, respectively. To investigate the effect of tryptone on iturinW production, 1% tryptone was added to the NB medium, and the culture conditions remained the same. *Escherichia coli* DH5α, used in this study, was cultured in LB medium and incubated at 37°C.

### Whole-genome sequencing, assembly, and annotation

To obtain the genome of *Bacillus* sp. wsm-1, the strain was incubated in NB medium at 28°C for 24 h. Genomic DNA was then extracted using a bacterial genomic DNA extraction kit (Tiangen, China) according to the manufacturer’s instructions. The DNA library was constructed using both Illumina NovaSeq platform at the Novogene and Nanopore Promethion platform and (Tianjin, China). The Illumina sequencing libraries were prepared using the NEBNext Ultra DNA Library Prep Kit (NEB, USA), with 350 bp fragmentation via sonication. Sequencing was performed on the Illumina PE150 platform (Illumina, USA). The Oxford Nanopore (ONT) libraries were constructed using the SQK-LSK114 ligation kit (Oxford Nanopore Technologies, UK). DNA shearing was carried out with a Covaris g-TUBE, and size selection was performed using the BluePippin System (Sage Science, USA) with a 0.75% agarose cassette (20–50 kb cutoff threshold). Sequencing was conducted on R10.4 flow cells (FLO-MIN114), generating data with an N50 of 14,634.0 bp. Base calling was performed using Guppy (v6.4.2) in high-accuracy mode, followed by adapter trimming with Guppy’s built-in functions. ONT read quality control was conducted using NanoPlot (v1.29.1). Genome assembly was conducted using Unicycler (v0.5.0), followed by three rounds of long-read polishing with Medaka (v1.2.0) and two rounds of short-read polishing with Pilon (v1.24), using default parameters for bacterial genomes. Circular genome confirmation was achieved by identifying overlapping regions (>1.5 kb, >95% identity) and adjusting the start site to the dnaA gene.

Gene prediction was performed using GeneMarkS-2+ (v2.5) integrated within the NCBI Prokaryotic Genome Annotation Pipeline (PGAP, release 2020-09-28). rRNAmmer software (v1.2) was used to predict rRNA, and tRNAscan software (v2.0.9) was used to predict the secondary structure of tRNA in tRNA region. Small RNAs (sRNAs) were identified using Rfam software, while prophages were predicted using phiSpy software. Repeat sequences were detected with RepeatMasker, and TR sequences were analyzed using TRF software. The prediction of secondary metabolite gene clusters in the *Bacillus* sp. wsm-1 genome was conducted using antiSMASH v5.1.

### Growth curve determination and iturin W production of *Bacillus* sp. wsm-1

To investigate the effect of tryptone on iturin W production, 1% tryptone was added to NB medium, and the culture conditions remained the same. Growth curves of *Bacillus* sp. wsm-1 in both NB and NB supplemented with 1% tryptone were measured by monitoring optical density at 600 nm (OD_600_) at regular intervals to assess bacterial growth phases. The production of iturin W was detected as we previously reported (16). Briefly, the cultures were incubated at 28°C with agitation at 150 rpm, and the cell-free supernatants were collected by centrifugation at different incubation time and then acidified to pH 2.5 using 6 M HCl. The crude iturin W was extracted with methanol and further purified and monitored by reversed-phase high-performance liquid chromatography (RP-HPLC; Agilent 1260, USA) using an Eclipse XDB-C18 column as we previously reported (16).

### RNA isolation, cDNA library construction, and sequencing

Transcriptomic analysis was performed by Novogene (Tianjin, China). To investigate the effect of tryptone on iturin W production, *Bacillus* sp. wsm-1 was cultured in NB medium, with or without 1% tryptone, and incubated for 24 h and 48 h, respectively. Total RNA was extracted using TRIzol reagent (Invitrogen, USA) following the manufacturer’s protocol, and the RNA contamination was removed using the MEGA clear Kit (Life Technologies, USA). rRNA depletion was performed via probe hybridization to enrich mRNA, and the enriched mRNA was then fragmented using divalent cations, followed by first-strand cDNA synthesis with random primers (M-MuLV reverse transcriptase) and second-strand synthesis using dUTP-containing dNTPs for strand specificity. RNA degradation and contamination were assessed using 1% agarose gel, while RNA integrity quantity was measure using an Agilent 2100 bioanalyzer (Agilent Technologies, USA).

A total amount of 3 µg of RNA per sample was used as input for RNA sample preparations. mRNA was obtained by removing the ribosomal RNA (rRNA) from the total RNA. Fragmentation buffer was added to randomly break the obtained mRNA into short fragments, and the library was constructed according to the method of strand- specific library construction. After construction, the Qubit2.0 fluorometer was used for initial quantification, followed by the Agilent 2100 bioanalyzer to assess the library insert size. Once the library passed quality control, different libraries were pooled according to the required effective concentration and target data amount, and Illumina sequencing was performed.

### Data analysis

The raw sequencing data initially contained adapter-contaminated reads and low-quality sequences. To ensure the accuracy of downstream analysis, we processed the raw reads using fastp (v0.20.0) to remove adapter contamination, poly-N sequences, and reads below a quality threshold of Q20. For functional annotation and quality control, reference genome and gene model annotation files were downloaded directly from NCBI database. Bowtie2 (v2.3.4.3) was used to align the filtered sequences to the reference genome, while featureCounts (v1.5.0-p3) from the Subread software package (http://subread.sourceforge.net) was employed to quantify the number of reads mapped to each gene. Finally, the FPKM (Fragments Per Kilobase of exon per Million fragments mapped) for each gene was calculated based on the gene length and the read count mapped to that gene (33).

The significance of differential gene expression was analyzed using edgeR v3.24.3, with the following steps: Filtering of low-expression genes (genes with counts per million [CPM] <1 in ≥2 samples), TMM normalization for library size adjustment, Quasi-likelihood *F*-tests with a false discovery rate (FDR) < 0.05, and Negative binomial distribution for *P*-value calculation. A corrected *P*-value threshold of 0.005 and log_2_(fold change) of 1 were applied to identify significantly differentially expressed genes. Gene Ontology (GO) functional enrichment analysis and KEGG pathway enrichment analysis were performed using clusterProfiler software. For both GO and KEGG enrichment analyses, a threshold of *padj* < 0.05 was applied to determine significant enrichment. Default parameters were used except where otherwise noted.

### Preparation of *Bacillus* sp. wsm-1 competent cells and transformation

The preparation of competent cells of *Bacillus* sp. wsm-1 was performed according to previous report with slight modification (34). Briefly, a single colony of *Bacillus* sp. wsm-1 was inoculated into starvation medium 1 (SM1) and cultured at 28°C with agitation at 200 rpm for 9–12 h. The seed culture was then transferred into 5 mL of fresh SM1 medium to achieve a final cell concentration of 0.5 at OD_600_. This culture was further incubated at 28°C with agitation at 200 rpm for 3 h. Afterward, an equal volume of starvation medium 2 (SM2) was added, and the cultures were incubated at 28°C with agitation at 200 rpm for an additional 2 h to prepare the competent cells.

To transform the plasmid into the prepared competent cells, 500 µL of competent cells and plasmid DNA (final concentration of 100 µg/mL) were mixed in a 1.5 mL centrifuge tube and incubated at 28°C with agitation at 200 rpm for 30 min. After incubation, 500 µL of fresh LB medium was added, and the mixture was further incubated with agitation for an additional 30 min. Subsequently, 200 µL of *Bacillus* sp. wsm-1 cell suspension was spread on LB agar plates containing selective antibiotics and incubated at 28°C for 48 h.

### Quantitative real-time reverse transcription PCR analysis

In order to validate the transcriptomic analysis, the qRT-PCR analysis was performed. *Bacillus* sp. wsm-1 was cultured in NB medium and NB supplemented with 1% tryptone for 24 h and 48 h, respectively. Total RNA was isolated from bacterial cell pellets using a standard TRIzol-based protocol. Subsequent cDNA synthesis was performed via reverse transcription using the HiScript II Q RT SuperMix for qPCR (+gDNA wiper) kit, following the manufacturer’s instructions, which included a genomic DNA removal step. qRT-PCR was carried out using SYBR Green Realtime PCR Master Mix (TOYOBO, Osaka, Japan) on a QuantStudio 6 Flex Real-Time PCR System (Thermo Fisher Scientific, USA). Primers were designed with Primer-BLAST, and the corresponding primer sequences are listed in Table 3. The specificity of the amplification was confirmed by melt curve analysis, which showed single peaks for all primer sets. The 16S rRNA gene of *Bacillus* sp. wsm-1 was used as the internal reference gene. The relative expression of the target gene was calculated using the comparative 2^−ΔΔCt^ method. All reactions were performed in three technical replicates for each sample.

**TABLE 3:** qRT-PCR primers sequences used in this study

Primer	Sequence (5′−3′)
RT16s-F	ACGGTCGCAAGACTGAAACT
RT16s-R	CCCAACATCTCACGACACGA
RTituW-A-F	CGCATGGTTCGATTCGGTTC
RTituW-A-R	TCCGGAATAAATCCGCCTCG
RTituW-D-F	TGTTTGAAGAAGCGAGCGATG
RTituW-D-R	TTCGCCTAAGCTATGGCCTG
RT04215-F	CGGGACATTGCGTGTATGAC
RT04215-R	CCGTACCAGCCGACAGTATT
RTglsA-F	GGCGCTCGTACTGATGGAT
RTglsA-R	CAGGTAATCCAGGCGGTCTT
RT09195-F	GGCAAGCCTTATCATTGCGG
RT09195-R	GTCTTGCTGAGGGTGAGCTT
RT11070-F	GGCGTCCTTCCTGACTTTA
RT11070-R	TGATTCGGCTTTCTCCCCAA
RT14295-F	GCCGGAAATGTCCACGAAAG
RT14295-R	CGTAAAGCGTGTCCGTTGAC
RT04210-F	TTTATGCGGATGCGGCAAAG
RT04210-R	GCCGCTTTTCCATGCAATCT
RTspoIIIAA-F	CGGGAGAATGGAAGACTCACA
RTspoIIIAA-R	CCGTACATCCTGCTGATCGT
RTcotJC-F	CCTGACAAGGTGATTGGCCT
RTcotJC-R	AAGACGCTGTGAACGGAACT
RTsigK-F	TAATGCCTTTCCACAACCGC
RTsigK-R	TCAGCCCGATTGTACCGATG

### Construction of the *ituW-A* mutant

To knock out gene *ituW-A,* upstream and downstream DNA fragments, approximately 800 bp in length, were amplified from the *Bacillus* sp. wsm-1 genome using primers pA1/pA2 and pA3/pA4 (Table 4). These fragments were then ligated to either sides of the chloramphenicol resistance cassette (CAT) in the suicide plasmid pKSV7, which does not replicate at 42°C. The constructed plasmid was subsequently transformed into *Bacillus* sp. wsm-1 competent cells and incubated on LB agar plates containing 15 µg/mL of chloramphenicol at 28°C. To obtain the *ituW-A* knockout strain, the resulting positive colonies were plated on LB agar plates containing chloramphenicol (15 µg/mL) and incubated at 42°C. The colonies with the *ituW-A* gene knockout were further verified using primers pA1 and pA4.

**TABLE 4:** Sequences of primers used for gene knockout and overexpression

Primer	Sequence (5′−3′)
pA1	TGCTCTAGATACGTAAACCTTGCAAGAAATATGTTCGAAAGTGTC
pA2	CATAAGCTTAGGTCCCCTCCTGGG
pA3	CGCGGATCCACCGAAAGATATCGCCATTATCGGG
pA4	CGCGAGCTCCTTAAGGATTCAGCGCGCTTCCCTTG
pMarA-F	GATGGATAAGGTTGATGAAGCCT
pMarA-R	TGAGTTAGCTCACTCATTAGGC
17,575F	ACGCGTCGACATGAAATTGAAGTATAAATTACTTATAGTCTGTTTTG
17,575R	TGCACTGCAGTTTTTATTAGATCATGGCGCGTTCAGA
14,295F	CGCGGATCCATGAAACAGAAATTTTTCTCAACTGT
14,925R	GACGTCGACTTTTTATTAGCAGTCGCAAGGC
09,195F	CGCGGATCCATGACGGCAGGCCTATCGATT
09,195R	GACGTCGACTTTTTATCAGCCAATAACAACCGGAAGCTC
11,070F	CGCGGATCCATGACCAGTTTAACAAAAATCAGAC
11,070R	TGCACTGCAGTTTTTATCAAAATTCAACAAGCAGCC
04,215F	ACGGTCGACATGAAGACAGTTTCCAAACG
04,215R	TGCACTGCAGTTTTTATTATTGATTTGACCAATATTGAATGTTGTCG
09,530F	CTAGTCTAGAATGAAGAAGACGATTATGTCCTTTGTCGC
09,530R	TGCACTGCAGTTTTACTAGTCTAATACTTTAACGTTTACTGTTCTGACGCCC
04,210F	ACGCGTCGACGTGTATTCGACAACATTCAATATTGGTCAAATCA
04,210R	TGCACTGCAGTTTTTATCAGTGTTTCAATGTAATGCTGATCACCG
11070-F2	AGAAAAGAGGAAGGAAATAATAAATGACCAGTTTAACAAAAATCAGACAGC
11070-R2	TAGACATCTAAATCTAGGTATCAAAATTCAACAAGCAGCCTGTC
09195-F2	GAAAAGAGGAAGGAAATAATAAATGACGGCAGGCCTATCGAT
09195-R2	TTAGACATCTAAATCTAGGTATCAGCCAATAACAACCGGAAGCT

### Overexpression of significantly changed DEGs

To overexpress the significantly altered DEGs, the *Himar1* gene was first deleted from plasmid pMarA, creating the plasmid pMarAΔ*Himar1*. The genes to be overexpressed were then amplified by PCR using the corresponding primers (Table 4) and ligated into pMarAΔ*Himar1* next to the PA promoter. To overexpress a second gene, it was amplified by PCR using primers from Table 4 and used to replace the kanamycin resistance gene in the first gene overexpression plasmid. The resulting plasmids were transformed into *Bacillus* sp. wsm-1 competent cells, plated on LB agar plates containing 5 µg/mL erythromycin, and incubated at 28°C. The positive colonies were further verified using primers pMarA-F/pMarA-R.

### Production detection of the iturin W

To investigate the relationship between DEGs and iturin W production under tryptone supplementation, the wild-type strain *Bacillus* sp. wsm-1, the *ituW-A* knockout strain, and the DEGs overexpression strains were cultured in NB medium with or without tryptone supplementation. The cultures were incubated at 28°C for 48 h with agitation at 150 rpm. After incubation, the cells were collected with centrifugation, and the supernatants were acidified to pH 2.5 using 6 M HCl. Then, resulting precipitate was then extracted with methanol and further purified by reversed-phase high-performance liquid chromatography (RP-HPLC; Agilent 1260, USA) using an Eclipse XDB-C18 column as we previously reported (16).

## Data Availability

The complete genomic sequence of *Bacillus* sp. wsm-1 has been deposited in NCBI database under GenBank accession number CP068989.1. The RNA-seq data have been submitted to Sequence Read Archive under BioProject accession numbers PRJNA725614, with the following SRA accession numbers: SRX10762445, SRX10762447, SRX10762448, and SRX10762450.
